# *Lactobacillus frumenti* Facilitates Intestinal Epithelial Barrier Function Maintenance in Early-Weaned Piglets

**DOI:** 10.3389/fmicb.2018.00897

**Published:** 2018-05-11

**Authors:** Jun Hu, Lingli Chen, Wenyong Zheng, Min Shi, Liu Liu, Chunlin Xie, Xinkai Wang, Yaorong Niu, Qiliang Hou, Xiaofan Xu, Baoyang Xu, Yimei Tang, Shuyi Zhou, Yiqin Yan, Tao Yang, Libao Ma, Xianghua Yan

**Affiliations:** ^1^State Key Laboratory of Agricultural Microbiology, College of Animal Sciences and Technology, Huazhong Agricultural University, Wuhan, China; ^2^The Cooperative Innovation Center for Sustainable Pig Production, Wuhan, China; ^3^Hubei Provincial Engineering Laboratory for Pig Precision Feeding and Feed Safety Technology, Wuhan, China

**Keywords:** *Lactobacillus frumenti*, early-weaned piglets, intestinal epithelial barrier function, intestinal microbiota, PICRUSt

## Abstract

Increased intestinal epithelial barrier function damages caused by early weaning stress have adverse effects on swine health and feed utilization efficiency. Probiotics have emerged as the promising antibiotic alternatives used for intestinal barrier function damage prevention. Our previous data showed that *Lactobacillus frumenti* was identified as a predominant *Lactobacillus* in the intestinal microbiota of weaned piglets. However, whether the intestinal epithelial barrier function in piglets was regulated by *L. frumenti* is still unclear. Here, piglets received a PBS vehicle or PBS suspension (2 ml, 10^8^ CFU/ml) containing the *L. frumenti* by oral gavage once a day during the period of 6–20 days of age prior to early weaning. Our data demonstrated that oral administration of *L. frumenti* significantly improved the intestinal mucosal integrity and decreased the serum endotoxin and D-lactic acid levels in early-weaned piglets (26 days of age). The intestinal tight junction proteins (including ZO-1, Occludin, and Claudin-1) were significantly up-regulated by *L. frumenti* administration. The serum immunoglobulin G (IgG) levels, intestinal secretory immunoglobulin A (sIgA) levels, and interferon-γ (IFN-γ) levels were significantly increased by *L. frumenti* administration. Furthermore, our data revealed that oral administration of *L. frumenti* significantly increased the relative abundances of health-promoting microbes (including *L. frumenti*, *Lactobacillus gasseri* LA39, *Parabacteroides distasonis*, and *Kazachstania telluris*) and decreased the relative abundances of opportunistic pathogens (including *Desulfovibrio desulfuricans* and *Candida humilis*). Functional alteration of the intestinal bacterial community by *L. frumenti* administration was characterized by the significantly increased fatty acids and protein metabolism and decreased diseases-associated metabolic pathways. These findings suggest that *L. frumenti* facilitates intestinal epithelial barrier function maintenance in early-weaned piglets and may be a promising antibiotic alternative used for intestinal epithelial barrier function damage prevention in mammals.

## Introduction

The intestinal epithelial barrier function is vital to animal and human health (Odenwald and Turner, 2017). The intestinal epithelial physical barrier (especially the intestinal tight junctions) confers the direct property of selective permeability to the intestinal epithelium (Steed et al., 2010; Suzuki, 2013; Zihni et al., 2016). The intestinal mucus layer forms defensive chemical barrier to protect intestinal epithelium from pathogens invasion (Corfield et al., 2000; McGuckin et al., 2011; Johansson and Hansson, 2016). The intestinal immunological barrier provides the important anti-infection roles for host (Rescigno, 2011). Intestinal microbiota-mediated pathogen resistance and beneficial functions for host suggest a critical role of intestinal microbial barrier function (Foxx-Orenstein and Chey, 2012; Buffie and Pamer, 2013). However, the intestinal epithelial barrier function disorders have been demonstrated be associated with several diseases, including inflammatory bowel diseases (IBD), irritable bowel syndrome (IBS), and infectious diarrhea (Turner, 2009).

Early weaning technology which contributes to shorten the cycle of slaughter in pigs and improve reproductive performance in sows has been widely used in pig production (Campbell et al., 2013). However, early weaning-induced stress will impair the intestinal epithelial barrier function, thereby increasing the risk of intestinal disorders, including intestinal inflammatory and diarrhea (Smith et al., 2010; Hu et al., 2013). Currently, antibiotics play important roles in the prevention for early weaning stress-induced intestinal epithelial barrier function damage in piglets (Lalles et al., 2007). However, the use of antibiotics in livestock farming was gradually banned (Casewell et al., 2003) due to that antibiotics-induced spread of antibiotic-resistant pathogens and antibiotic residues in foods have emerged as serious problems worldwide (Allen et al., 2014). Thus, finding alternatives to antibiotics used in early weaning stress-induced intestinal epithelial barrier function damage prevention is important for livestock farming and food safety.

Growing evidences have revealed that probiotics (benign commensals) facilitated the intestinal epithelial barrier function maintenance in mammals (Bron et al., 2011, 2017; Wan et al., 2016). Given that *Lactobacillus* confer the health-promoting roles in mammals (Kleerebezem et al., 2010; van Baarlen et al., 2013), we screened the *Lactobacillus* in our previous data and found that *Lactobacillus frumenti* was identified as a predominant *Lactobacillus* in the intestinal microbiota of weaned piglets (Hu et al., 2016). However, whether the *L. frumenti* regulates intestinal epithelial barrier function in early-weaned piglets has not been explored. This study was conducted to explore to the potential regulatory role of *L. frumenti* in the intestinal epithelial barrier function in early-weaned piglets. Our findings revealed an important role for *L. frumenti* in intestinal epithelial barrier function improvement in early-weaned piglets. Our data suggest that *L. frumenti* may be a potential antibiotic alternative used for intestinal epithelial barrier function damage prevention in mammals.

## Materials and Methods

### Animals and Sample Collection

*Lactobacillus frumenti* (JCM 11122) used in this study was obtained from Japan Collection of Microorganisms. *L. frumenti* was cultured in de Man, Rogosa and Sharpe (MRS) medium at 37°C as previously described (Muller et al., 2000). A total of 100 crossbred piglets (Landrace × Yorkshire) with similar birth weight were randomly assigned to 2 groups. Piglets in the same group were then randomly assigned to 5 pens and each pen contained 10 piglets. All piglets in group 1 (Control, Ctrl) received a vehicle (sterile PBS, 2 ml) by oral gavage once a day during the period of 6–20 days of age. All piglets in group 2 (*L. frumenti*, LF) received a PBS suspension (2 ml, 10^8^ CFU/ml) containing the *L. frumenti* by oral gavage once a day during the period of 6–20 days of age. All the piglets were early-weaned at 21 days of age. At 26 days of age, one piglet was randomly selected from each pen and weighted and then slaughtered for samples collection. All piglets had free access to diet and water during the entire experimental period. The diet compositions in detail for early-weaned piglets was shown in Supplementary Table S1. The serums were collected from these 10 piglets slaughtered for serum endotoxin, D-lactic acid, immunoglobulin G (IgG), immunoglobulin A (IgA), and immunoglobulin M (IgM) levels measurements. To reduce sample variability, the intestinal segments samples were collected from the approximately middle positions in the piglet intestinal tracts (including duodenum, jejunum, and ileum), respectively, as previously described (Hu et al., 2017). The duodenum, jejunum, and ileum segments obtained (approximately 2-cm in length) were rinsed with PBS to remove intestinal contents and then fixed in 4% paraformaldehyde for analysis of intestinal morphology, periodic acid schiff (PAS) stain, and immunohistochemistry. The intestinal tracts (including duodenum, jejunum, and ileum) (approximately 5-cm in length) were rinsed with PBS to remove intestinal contents and then stored at -80°C prior to further analysis for protein expressions, relative mRNA expressions of intestinal tissues, secretory IgA levels, and intestinal cytokines levels. The immune organs (including spleen and thymus) were collected for the weights of immune organs measurement. Fresh feces were individually collected from the rectums of piglets slaughtered. A total of 10 fresh feces samples individually collected were frozen in liquid nitrogen immediately and then stored at -80°C before fecal microbial genomic DNA extraction. Piglets handling protocols (permit number: HZAUSW2013-0006) were approved by the Institutional Animal Care and Use Committee of Huazhong Agricultural University. The methods were carried out in accordance with the approved guidelines.

### Morphological Analysis of Intestinal Epithelial Tissues of the Early-Weaned Piglets

Haematoxylin and Eosin (H&E) staining was performed to analyze intestinal morphology using our previously described protocol (Hu et al., 2017). Briefly, following removal from the 4% paraformaldehyde solution, the duodenum, jejunum, and ileum segments were washed and then embedded in paraffin. Subsequently, these segments were cut into approximately 5-μm thick sections then stained with haematoxylin, followed by eosin. Digital images of intestinal morphology at 40× and 100× magnification were obtained using a light microscope. The villi heights and crypts depths of intestinal segments were measured using Image-Pro Plus software (version 6.0) based on at least 50 representative, well oriented villi and crypts per piglet in a blinded manner, as described previously (Jeppesen et al., 2005; Igarashi and Guarente, 2016). For each intestinal segment, villus height was defined as the distance from the crypt-villus junction to the tip of the villus and crypt depth was defined as the distance from the bottom of the crypt to the crypt-villus junction, as described previously (Igarashi and Guarente, 2016; Hu et al., 2017).

### Measurement of Serum Endotoxin and D-Lactic Acid Levels

The endotoxin and D-lactic acid levels in the serum were measured using the endotoxin (Nanjing Jiancheng Bioengineering Institute of China, H178) and D-lactic acid (Nanjing Jiancheng Bioengineering Institute of China, H263) ELISA assay kits according to the manufacturer’s protocol, respectively.

### Western Blot Analysis of Intestinal Tight Junction Proteins Expression

The whole cell lysates (WCLs) of intestinal segments (including duodenum, jejunum, and ileum) used for western blotting were prepared using lysis buffer (50 mM Tris, 150 mM NaCl, 1 mM EDTA, 1% Triton X-100, 1 μg/mL pepstatin, 1 μg/mL aprotinin, 1 μg/mL leupeptin, and 1 mM PMSF), respectively. Western blot assay was performed using our previously described protocol (Hu et al., 2017). Briefly, protein extracts were subjected to sodium dodecyl sulfate polyacrylamide gel electrophoresis (SDS-PAGE) using 6% or 12% polyacrylamide gels and then transferred to polyvinylidene fluoride (PDVF) membranes. Subsequently, PVDF membranes were incubated in Tris-buffered saline (TBS) buffer containing 0.1% Tween-20 and 5% fat-free milk for 1 h, followed by incubation with a specific primary antibody overnight at 4°C. The PVDF membranes were incubated with horseradish peroxidase (HRP)-conjugated secondary antibodies for 1.5 h at room temperature. Finally, the signals were detected using an enzyme-linked enhanced chemiluminescence (ECL) reagent (Thermo Scientific, 34080) according to the manufacturer’s protocol. The following antibodies were used in the western blot assays: HRP-conjugated secondary antibodies [Santa Cruz Biotechnology, goat anti-rabbit (sc-2004)], JAM1 antibody (ABclonal Technology, A1241), ZO-1 antibody (ABclonal Technology, A11417), Occludin antibody (ABclonal Technology, A2601), Claudin-1 antibody (ABclonal Technology, A2196), Mucin 2 (Abcam, ab11197), and β-actin (ABclonal Technology, AC026).

### Immunohistochemistry Analysis of Intestinal Tight Junction Proteins

Immunohistochemistry was performed to analyze intestinal tight junction proteins expression distribution in piglets. We used the specific rabbit monoclonal antibody to conduct this immunohistochemistry assay. The following antibodies were used in the immunohistochemistry assay: Claudin-1 antibody (Abcam, ab211737), Occludin antibody (Abcam, ab216327), and ZO-1 antibody (Abcam, ab214228). The immunohistochemistry assay was performed using the manufacturer’s protocol from Abcam. Digital images of intestinal morphology at 100× and 200× magnification were obtained using a light microscope.

### Measurement of pH Value in Intestinal Contents

Intestinal contents in segments (including duodenum, jejunum, and ileum) were collected in the sterilized centrifuge tubes, respectively. The pH values of intestinal contents were measured using a mini-pH meter as previously described (Xiong et al., 2015).

### Measurement of Relative mRNA Expression of Intestinal Secretory Mucins Proteins

Given that mucins proteins were secreted from the intestinal epithelium and formed the mucous layer barrier, we used the relative mRNA expression of intestinal secretory mucins proteins in intestinal segments to evaluate the expression levels of mucins proteins as previously described (Ren et al., 2010; Pieper et al., 2012). Total RNA was extracted from the intestinal segments (including duodenum, jejunum, and ileum) using TRIzol reagent (Invitrogen, 15596), respectively. The cDNA was generated for Quantitative Real-Time PCR (qRT-PCR) validation using the PrimeScript TM RT reagent Kit with gDNA Eraser (Takara, RR047A). The relative mRNA expression of intestinal secretory mucins proteins (including mucin 1, mucin 2, mucin 4, mucin 13, and mucin 20) were measured by qRT-PCR with GAPDH as an internal reference. The specific primers for qRT-PCR were listed in **Table 1** below. qRT-PCR was carried out with Bio-Rad CFX384 using iTaq^TM^ Universal SYBR^^®^^ Green Supermix (Bio-Rad, 1725124). The amplification procedures were 95°C for 30 s initially, followed by 40 cycles of 95°C for 5 s, 60°C for 15 s, and 72°C for 15 s. The relative gene expression analysis was performed using the 2^-ΔΔ^*^C^*^t^ method.

**Table 1:** Specific primers for GAPDH and mucins genes.

GAPDH	Forward	5′-CCTTCATTGACCTCCACTACAT-3′
	Reverse	5′-GGATCTCGCTCCTGGAAGA-3′
Mucin 1	Forward	5′-GTGCCGACGAAAGAACTG-3′
	Reverse	5′-TGCCAGGTTCGAGTAAGAG-3′
Mucin 2	Forward	5′-CTGTGTGGGGCCTGACAA-3′
	Reverse	5′-AGTGCTTGCAGTCGAACTCA-3′
Mucin 4	Forward	5′-TTCACTCCAACCATCCTTCCA-3′
	Reverse	5′-CTCGTTCCACTTGTCTGTTCC-3′
Mucin 13	Forward	5′-GCTACAGTGGAGTTGGCTGT-3′
	Reverse	5′-GACGAATGCAATCACCAGGC-3′
Mucin 20	Forward	5′-AGGCAGTTACAACATCCACAGAAG-3′
	Reverse	5′-CTGTAGACCATGGCCGAGAAC-3′

### Measurement of Intestinal Goblet Cells Numbers

The intestinal goblet cells numbers were measured by intestinal periodic acid schiff (PAS) stain. Briefly, following removal from the 4% paraformaldehyde solution, the duodenum, jejunum, and ileum segments were washed and then embedded in paraffin. Subsequently, these segments were cut into approximately 5-μm thick sections then stained by PAS stain kit (Abcam, ab150680) according to the manufacturer’s protocol. The intestinal goblet cells were stained with magenta color. Digital images of intestinal morphology at 100× and 200× magnification were obtained using a light microscope. The numbers of intestinal goblet cells were normalized to the villus-crypt units.

### Measurements of Body Weights, Weights of Immune Organs, Serum IgG/IgM/IgA Levels, Intestinal Secretory IgA Levels, and Intestinal Cytokines Levels

The immune organs (including spleen and thymus) collected were weighed, respectively. The weights of immune organs were normalized to body weight of piglets. The serum IgG/IgM/IgA levels were measured using the IgG (Nanjing Jiancheng Bioengineering Institute of China, H106), IgM (Nanjing Jiancheng Bioengineering Institute of China, H109), and IgA (Nanjing Jiancheng Bioengineering Institute of China, H108) ELISA assay kits according to the manufacturer’s protocol, respectively. The intestinal secretory IgA (sIgA) levels in intestinal segments (including duodenum, jejunum, and ileum) were measured using the sIgA ELISA assay kit (Shanghai Sangon Biotech, D740102). The cytokines levels in intestinal segments (including duodenum, jejunum, and ileum) were measured using the interferon-γ (IFN-γ) ELISA assay kit (Shanghai Sangon Biotech, D740049), interleukin-6 (IL-6) ELISA assay kit (Shanghai Sangon Biotech, D740104), transforming growth factor-β1 (TGF-β1) ELISA assay kit (Shanghai Sangon Biotech, D740095), and tumor necrosis factor-α (TNF-α) ELISA assay kit (Shanghai Sangon Biotech, D740103), respectively. The protein content of the WCLs of intestinal epithelium was quantified using the Pierce BCA Protein Assay Kit (Thermo Scientific, 23227). The sIgA levels, IFN-γ levels, IL-6 levels, TGF-β1 levels, and TNF-α levels were all normalized to the total protein concentration (mg/L) of WCLs.

### Fecal Microbial Genomic DNA Extraction

Total microbial genomic DNA, including bacterial and fungal genomic DNA in the feces of piglets, was extracted using a combined method of cetyl trimethyl ammonium bromide (CTAB) and bead-beating as our previously described (Hu et al., 2016).

### Bacterial 16S rDNA Gene and Fungal ITS Gene High-Throughput Sequencing

V3–V4 region of bacterial 16S rDNA gene and ITS2 region of fungal ITS gene were amplified to construct DNA libraries for high-throughput sequencing, respectively, using our previously described methods with slight modifications (Hu et al., 2016). The primer sequences for V3–V4 region amplification were as follows: 5′–NNNNNNNNACTCCTACGGGAGGCAGCAG–3′ (forward) and 5′– GGACTACHVGGGTWTCTAAT–3′ (reverse). The primer sequences for ITS2 region amplification were as follows: 5′–NNNNNNNNGCATCGATGAAGAACGCAGC–3′ (forward) and 5′–TCCTCCGCTTATTGATATGC–3′ (reverse). The PCR primer barcodes facilitate the segregation of sequencing information output according to the sampling numbers. The Qualified libraries are sequenced pair end on the MiSeq System, with the sequencing strategy PE300 (PE301+8+8+301) (MiSeq Reagent Kit).

### Sequencing Data Analysis

In order to obtain more accurate and reliable results in subsequent bioinformatics analysis, the raw data from Illumina Miseq high-throughput sequencing will be pre-processed to eliminate the adapter pollution and low quality for obtaining clean reads by the following procedures: (1) those sequence reads not having an average quality of 30 over a 25 bp sliding window based on the phred algorithm were truncated and those trimmed reads having <60% of their original length, as well as its paired read, were also removed; (2) those reads contaminated by adapter (default parameter: 15 bases overlapped by reads and adapter with maximal 3 bases mismatch allowed) were removed; (3) those reads with ambiguous base (N base), and its paired reads were removed; (4) those reads with low complexity (default: reads with 10 consecutive same base) were removed.

The paired-end clean reads with overlap were merged to tags using Connecting Overlapped Pair-End (COPE, V1.2.1) software. Subsequently, bacterial tags were clustered into Operational Taxonomic Units (OTUs) at 97% sequence similarity by scripts of USEARCH (v7.0.1090) software. Bacterial OTU representative sequences were taxonomically classified by scripts of Ribosomal Database Project (RDP) Classifier v.2.2 software based on the Ribosomal Database Project (RDP) database. Fungal tags were clustered into OTUs at 97% sequence similarity by scripts of USEARCH (v7.0.1090) software. Fungal OTU representative sequences were taxonomically classified using RDP Classifier v.2.2 software based on the UNITE database. Venn diagram, which visually displays the numbers of common and unique OTUs among groups, was drawn by the package “VennDiagram” of R (v3.1.1) software. Principal component analysis (PCA) based on OTUs abundance was drawn by the package “ade4” of R (v3.1.1) software. Beta diversity based on weighted UniFrac distance was performed by QIIME (v1.80) software and displayed by the principal coordinates analysis (PCoA) and cluster tree analysis. Chao index, Shannon index, and Simpson index which reflect alpha diversity were calculated by Mothur (v1.31.2) and the corresponding rarefaction curve are drawn by R (v3.1.1) software. Heat maps were generated using the package “gplots” of R (v3.1.1) software. The distance algorithm was “Euclidean” and the clustering method was “complete.” Genus-level phylogenetic tree was constructed using the Quantitative Insights Into Microbial Ecology (QIIME) (v1.80) built-in scripts and was imaged by R (v3.1.1) software at last.

### Functional Profiles Analysis of Bacterial Community Using PICRUSt

Phylogenetic investigation of communities by reconstruction of unobserved states (PICRUSt) method was applied for predicting the gene family abundances of bacterial communities based on the 16S rDNA gene data and a database of reference genomes (Langille et al., 2013). Briefly, the PICRUSt which consisted of two steps: gene content inference and metagenome inference was performed as described previously (Langille et al., 2013).

### Statistical Analysis

Statistical analyses were carried out using GraphPad Prism (version 6.0c) software, R (v3.0.3) software, Metastats, and STAMP (Statistical Analysis of Metagenomic Profiles). Metastats software was used to identify the differentially abundant taxa (genera and species) among groups. After the statistical comparison of taxa, we used the Benjamini–Hochberg to control the false discovery rate using the package ‘p. adjust’ of R (v3.0.3) software. STAMP software was applied to detect the differentially abundant Kyoto Encyclopedia of Genes and Genomes (KEGG) pathways among groups with false discovery rate correction. All the data are shown as mean ± SEM. The Student’s *t*-tests were used for to compare differences between two groups. For all tests, differences were considered significant at a *p*-value < 0.05. Replicate numbers of the experiments performed are shown in the figure legends.

## Results

### Oral Administration of *L. frumenti* Improves the Intestinal Epithelial Barrier Function in Early-Weaned Piglets

**FIGURE 1:**
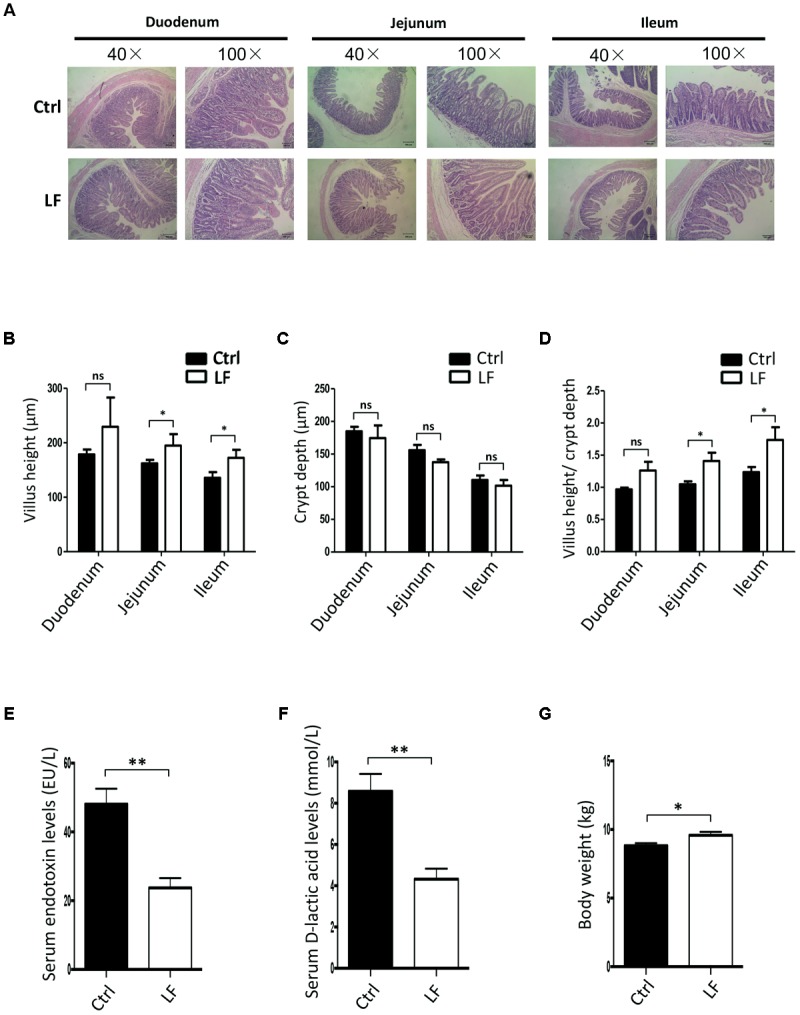
The effects of *Lactobacillus frumenti* on intestinal epithelial mucosal integrity and the levels of serum endotoxin and D-lactic acid. **(A)** Intestinal morphology shown by H&E staining of duodenum, jejunum, and ileum tissues of weaned piglets. The images of intestinal morphology at 40× magnification and 100× magnification were shown, respectively (Ctrl, Control; LF, *L. frumenti*). **(B–D)** Statistical analysis of villus height (μm) **(B)**, crypt depth (μm) **(C)**, and the ratios of villus height (μm) to crypt depth (μm) **(D)** in the duodenum, jejunum, and ileum of weaned piglets, respectively. Data are represented as mean ± SEM (*n* = 5; ^∗^*p* < 0.05, ns, not significant, *p* > 0.05). **(E)** The serum endotoxin levels of weaned piglets. Data are represented as mean ± SEM (*n* = 5; ^∗∗^*p* < 0.01). **(F)** The serum D-lactic acid levels of weaned piglets. Data are represented as mean ± SEM (*n* = 5; ^∗∗^*p* < 0.01). **(G)** The body weights of weaned piglets. Data are represented as mean ± SEM (*n* = 5; ^∗^*p* < 0.05).

The present study was aimed to investigate whether *L. frumenti* contributes to improve the intestinal epithelial barrier function in early-weaned piglets. The results demonstrated that beforehand oral administration of *L. frumenti* prior to early weaning significantly increased the villi heights and the ratios of villi heights to crypts depths in the jejunum and ileum in early-weaned piglets, respectively, suggesting an improved intestinal mucosal integrity and intestinal epithelial barrier function (**Figures 1A–D**). The levels of serum endotoxin and D-lactic acid, which are key indexes for intestinal epithelial barrier function evaluation, were both significantly decreased with oral administration of *L. frumenti* prior to early weaning in piglets (**Figures 1E,F**). Importantly, the body weights of early-weaned piglets were significantly increased by oral administration of *L. frumenti* (**Figure 1G**). These findings suggest that *L. frumenti* facilitates improve the intestinal epithelial barrier function in early-weaned piglets on a whole.

### The Effects of *L. frumenti* on the Intestinal Epithelial Physical Barrier, Chemical Barrier, and Immunological Barrier Functions in Early-Weaned Piglets

**FIGURE 2:**
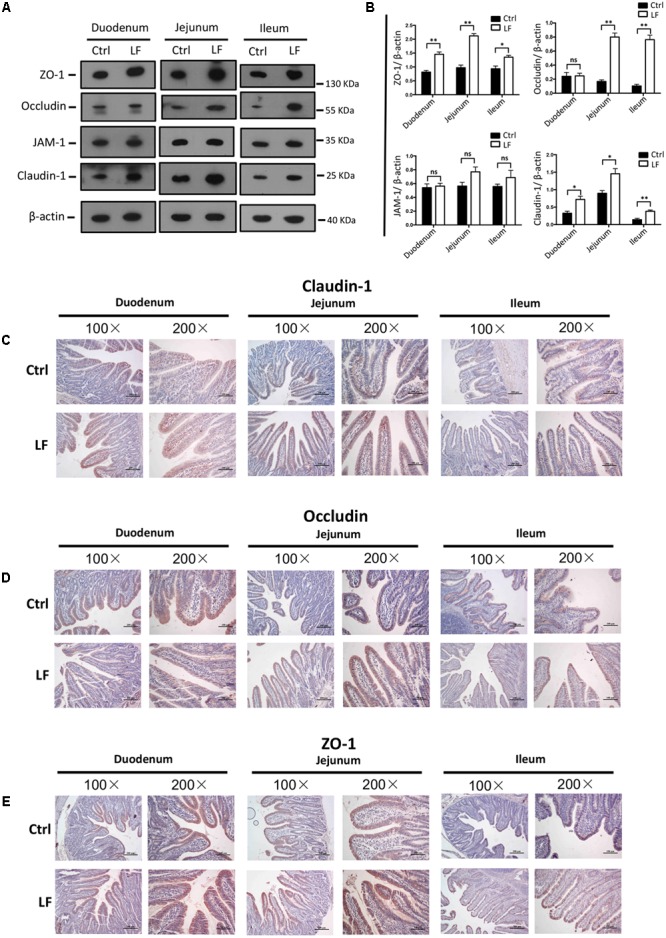
The effects of *L. frumenti* on the intestinal epithelial physical barrier functions in early-weaned piglets. **(A,B)** Western blotting measurements of the expression levels of tight junction proteins (including ZO-1, Occludin, JAM-1, and Claudin-1) and a housekeeping protein (β-actin) in the small intestinal tissues (including duodenum, jejunum, and ileum) from early-weaned piglets **(A)**. Normalization and quantitation of ZO-1/β-actin, Occludin/β-actin, JAM-1/β-actin, and Claudin-1/β-actin as prepared described in **(B)**. Data are shown as mean ± SEM for at least three different experiments (ns, not significant, *p* > 0.05, ^∗^*p* < 0.05, ^∗∗^*p* < 0.01) (Ctrl, Control; LF, *L. frumenti*). **(C–E)** Immunohistochemistry analysis of tight junction proteins [including Claudin-1 **(C)**, Occludin **(D)**, and ZO-1 **(E)**] in the small intestinal tissues (including duodenum, jejunum, and ileum) from early-weaned piglets. The images of intestinal morphology at 100× magnification and 200× magnification were shown, respectively.

**FIGURE 3:**
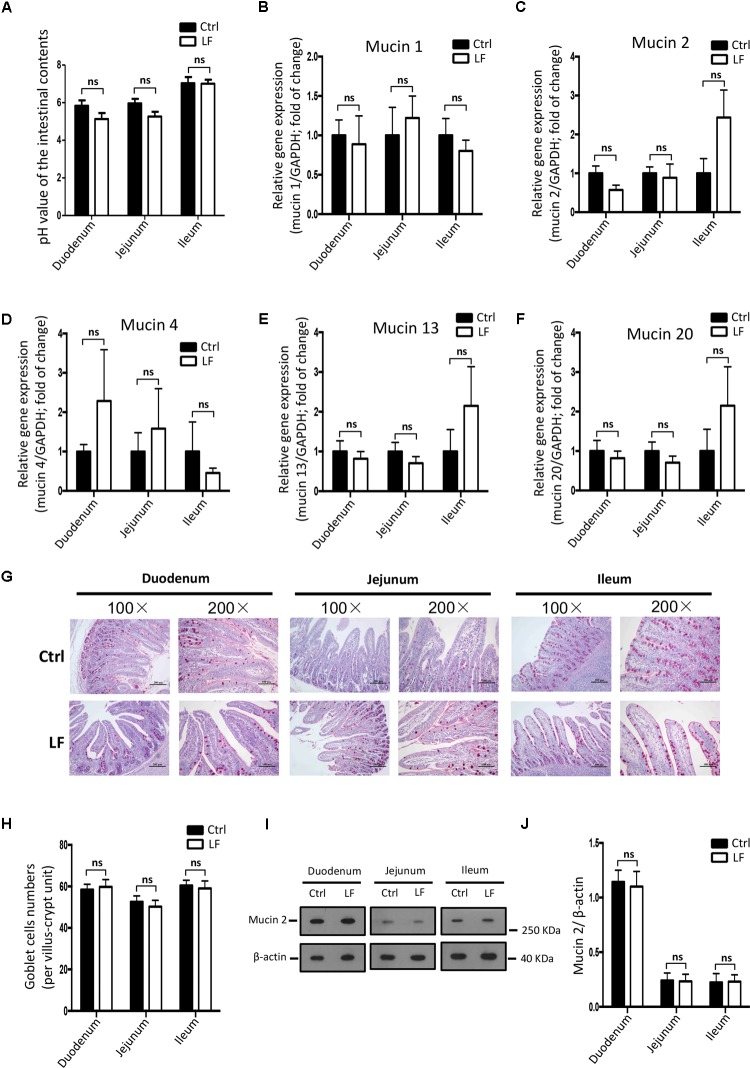
The effects of *L. frumenti* on the intestinal epithelial chemical barrier functions in early-weaned piglets. **(A)** The pH values of the intestinal contents in small intestinal tissues (including duodenum, jejunum, and ileum) from early-weaned piglets. Data are represented as mean ± SEM (*n* = 5; ns, not significant) (Ctrl, Control; LF, *L. frumenti*). **(B–F)** The relative mRNA expression of intestinal mucins proteins measured by Q-PCR. Normalization and quantitation of Mucin 1/GAPDH **(B)**, Mucin 2/GAPDH **(C)**, Mucin 4/GAPDH **(D)**, Mucin 13/GAPDH **(E)**, and Mucin 20/GAPDH **(F)** were shown in bar chart. Data are represented as mean ± SEM (*n* = 5; ns, not significant). **(G)** The intestinal goblet cells shown by PAS staining of duodenum, jejunum, and ileum tissues of weaned piglets. The images of intestinal morphology at 100× magnification and 200× magnification were shown, respectively. **(H)** The statistical analysis of intestinal goblet cells numbers in duodenum, jejunum, and ileum tissues of weaned piglets. Data are represented as mean ± SEM (*n* = 5; ns, not significant). **(I,J)** Western blotting measurements of the expression levels of mucin 2 protein and a housekeeping protein (β-actin) in the small intestinal tissues (including duodenum, jejunum, and ileum) from early-weaned piglets **(I)**. Normalization and quantitation of mucin 2/β-actin as prepared described in **(J)**. Data are shown as mean ± SEM for at least three different experiments (ns, not significant).

**FIGURE 4:**
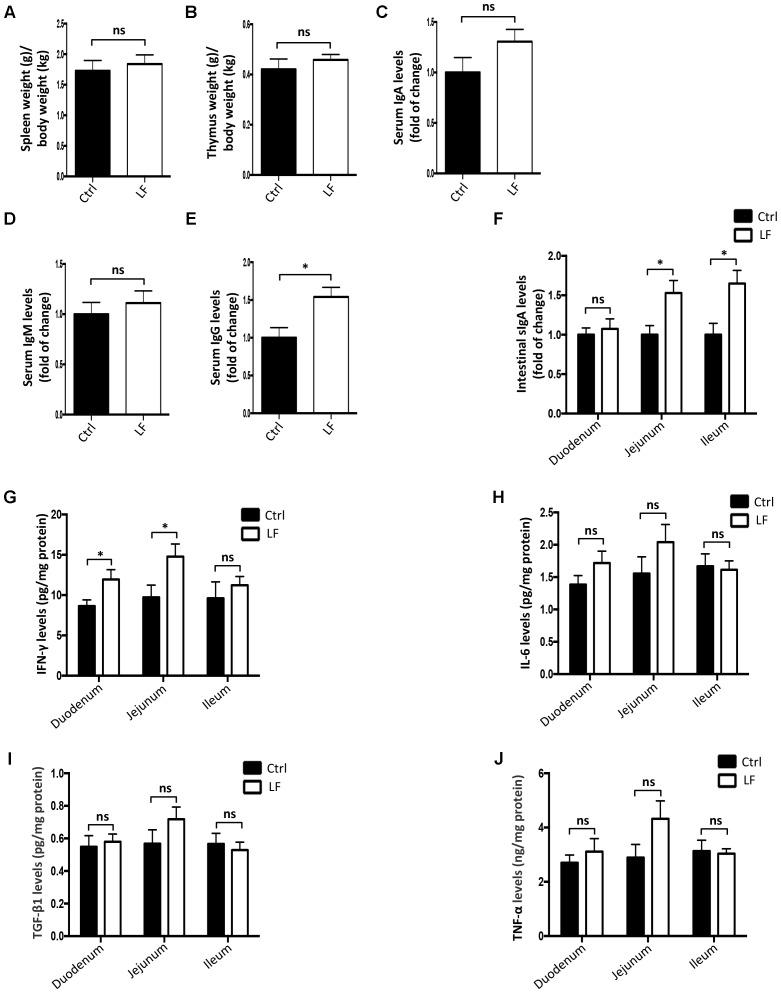
The effects of *L. frumenti* on the intestinal epithelial immunological barrier functions in early-weaned piglets. **(A,B)** The weights of immune organs (including spleen and thymus). The weights (g) of spleen **(A)** and thymus **(B)** were normalized to body weights (kg) of piglets. Data are represented as mean ± SEM (*n* = 5; ns, not significant) (Ctrl, Control; LF, *L. frumenti*). **(C–E)** The levels of serum IgA **(C)**, IgM **(D)**, and IgG **(E)**. Data are represented as mean ± SEM (*n* = 5; ns, not significant, *p* > 0.05, ^∗^*p* < 0.05). **(F)** The levels of intestinal secretory IgA in intestinal segments (including duodenum, jejunum, and ileum). Data are represented as mean ± SEM (*n* = 5; ns, not significant, *p* > 0.05, ^∗^*p* < 0.05). **(G–J)** The levels of cytokines [including IFN-γ **(G)**, IL-6 **(H)**, TGF-β1 **(I)**, and TNF-α **(J)**] in intestinal segments (including duodenum, jejunum, and ileum). Data are represented as mean ± SEM (*n* = 5; ns, not significant, *p* > 0.05, ^∗^*p* < 0.05).

We further investigated the role of *L. frumenti* on intestinal epithelial barrier function at several functional levels (physical barrier, chemical barrier, and immunological barrier). Our data demonstrated that the expression levels of tight junction proteins (including ZO-1, Occludin, and Claudin-1) in the small intestinal tissues (including duodenum, jejunum, and ileum) from early-weaned piglets were significantly increased with oral administration of *L. frumenti* (**Figures 2A,B**), suggesting an enhancement in intestinal epithelial physical barrier function. We further used immunohistochemistry assay to investigate the tight junction proteins expression distribution in the small intestinal tissues (including duodenum, jejunum, and ileum) of early-weaned piglets. The results showed that the expression distribution of tight junction proteins (including Claudin-1, Occludin, and ZO-1) was not altered by oral administration of *L. frumenti* (**Figures 2C–E**). To evaluate the effects of oral administration of *L. frumenti* on intestinal epithelial chemical barrier function in early-weaned piglets, we examined the pH values of small intestinal contents and relative mRNA expression levels of mucins (including mucin 1, mucin 2, mucin 4, mucin 13, and mucin 20) in small intestinal epithelial tissues. The results of this study indicated that the pH values of small intestinal contents (including duodenum, jejunum, and ileum) were not significantly altered by *L. frumenti* administration (**Figure 3A**). The relative mRNA expression levels of mucins (including mucin 1, mucin 2, mucin 4, mucin 13, and mucin 20) in small intestinal epithelial tissues (including duodenum, jejunum, and ileum) were also not significantly altered by *L. frumenti* administration (**Figures 3B–F**). The result of intestinal PAS stain indicated that the numbers of intestinal goblet cells (can secrete mucins proteins) were not significantly changed by *L. frumenti* administration (**Figures 3G,H**). The expression levels of mucin 2 protein were also not significantly altered by *L. frumenti* administration (**Figures 3I,J**). We further investigated the effects of oral administration of *L. frumenti* on the intestinal epithelial immunological barrier function in early-weaned piglets. The results demonstrated that oral administration of *L. frumenti* did not significantly change the weights of immune organs (including spleen and thymus) (**Figures 4A,B**) and the levels of serum immunoglobulin A (IgA) (**Figure 4C**) and immunoglobulin M (IgM) (**Figure 4D**) and in early-weaned piglets. Interestingly, the levels of serum immunoglobulin G (IgG) (**Figure 4E**) and intestinal secretory immunoglobulin A (sIgA) (**Figure 4F**) were both significantly increased by *L. frumenti* administration in early-weaned piglets. The levels of intestinal IL-6, TGF-β1, and TNF-α were not significantly altered, whereas the levels of intestinal IFN-γ were significantly increased by *L. frumenti* administration in early-weaned piglets (**Figures 4G–J**). These data revealed that oral administration of *L. frumenti* contributes to the intestinal mucosal immunity performed by sIgA, anti-infection immunity performed by IgG, and antiviral immunity performed by IFN-γ. These findings above suggest that *L. frumenti* facilitates improving the intestinal epithelial physical barrier and immunological barrier functions in early-weaned piglets.

### The Effects of *L.frumenti* on the Intestinal Microbial Communities in Early-Weaned Piglets

**FIGURE 5:**
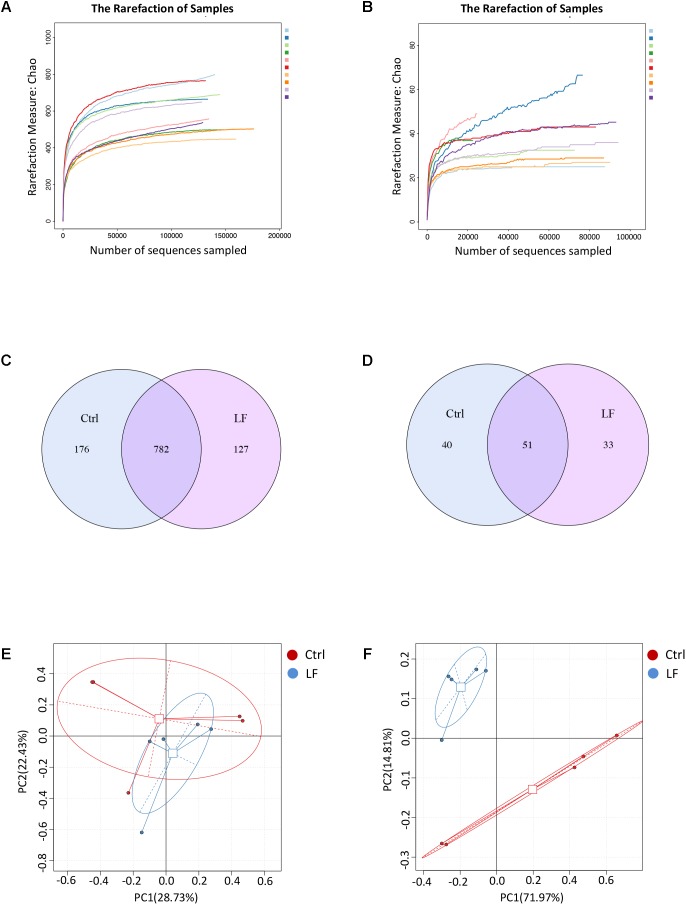
The shifts in intestinal microbial OTUs compositions in early-weaned piglets with oral administration of *L. frumenti*. **(A,B)** Bacterial rarefaction curves **(A)** and fungal rarefaction curves **(B)** based on Chao index were used to assess the sequencing depth for each sample. Each sample was distinguished by different colors of lines. **(C,D)** Venn diagrams for bacterial OTUs compositions **(C)** and fungal OTUs compositions **(D)** (Ctrl, Control; LF, *L. frumenti*). **(E,F)** Scatterplots from principal component analysis (PCA) of bacterial OTUs compositions **(E)** and fungal OTUs compositions **(F)** in each sample.

**FIGURE 6:**
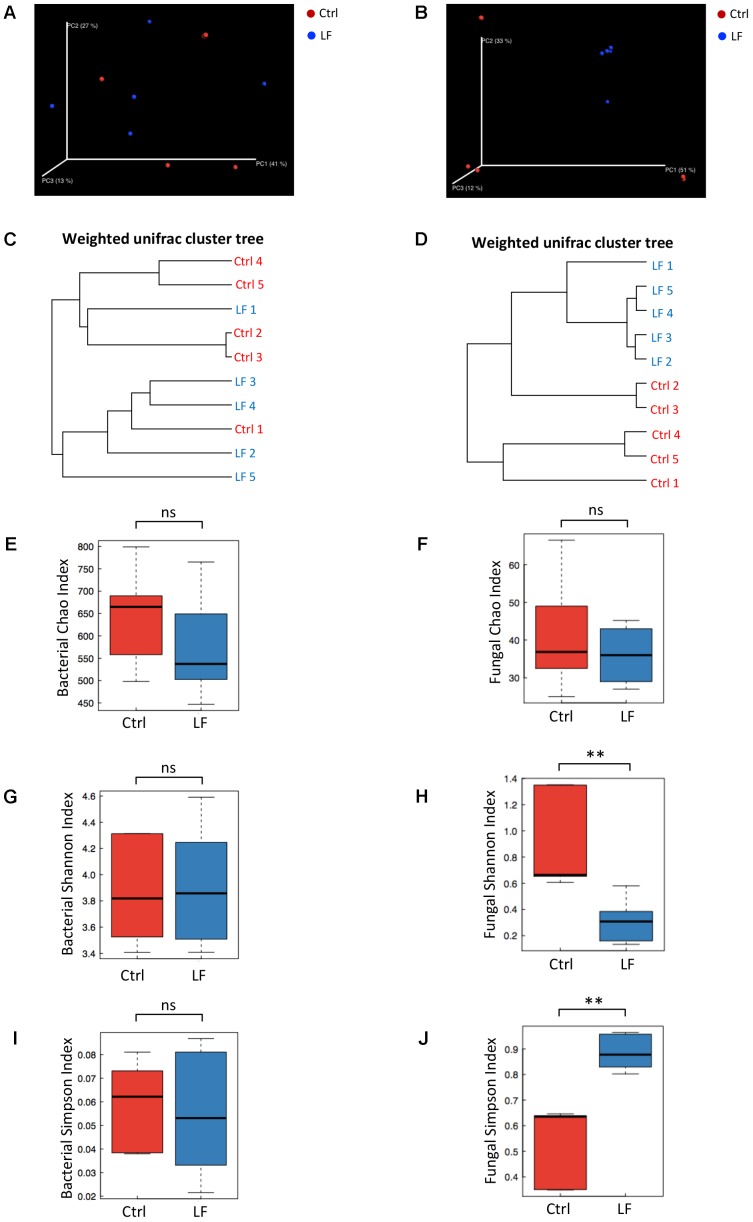
The changes of intestinal microbial diversities in early-weaned piglets with oral administration of *L. frumenti*. **(A,B)** Scatterplots from principal coordinates analysis (PCoA), based on weighted UniFrac distance in bacterial communities **(A)** and fungal communities **(B)** (Ctrl, Control; LF, *L. frumenti*). **(C,D)** Cluster tree analyses based on weighted UniFrac distance in bacterial communities **(C)** and fungal communities **(D)**. **(E,F)** The alpha diversities determined by Chao index in bacterial communities **(E)** and fungal communities **(F)**. Data are represented as mean ± SEM (*n* = 5; ns, not significant, *p* > 0.05). **(G,H)** The alpha diversities determined by Shannon index in bacterial communities **(G)** and fungal communities **(H)**. Data are represented as mean ± SEM (*n* = 5; ^∗∗^*p* < 0.01, ns, not significant, *p* > 0.05). **(I,J)** The alpha diversities determined by Simpson index in bacterial communities **(I)** and fungal communities **(J)**. Data are represented as mean ± SEM (*n* = 5; ^∗∗^*p* < 0.01, ns, not significant, *p* > 0.05).

We next investigated the effects of *L. frumenti* on the intestinal microbial communities in early-weaned piglets. We totally collected 1426,604 and 733,250 high-quality sequences of V3–V4 region and ITS2 region in 10 fecal samples from piglets after quality control, respectively. The average numbers of high-quality sequences generated per sample were 142,660 and 73,325 from bacterial and fungal populations, respectively. Rarefaction curves demonstrated that almost all the bacterial and fungal species were detected in feces of weaned piglets (**Figures 5A,B**). A total of 782 core OTUs in bacterial communities and 51 core OTUs in fungal populations were identified, respectively (**Figures 5C,D**). PCA based on the microbial OTUs compositions indicated that the samples in control (Ctrl) group separated with that in *L. frumenti* (LF) group, suggesting a shift in the intestinal microbial communities (especially the intestinal fungal communities) with the oral administration of *L. frumenti* (**Figures 5E,F**). PCoA based on the weighted UniFrac distances showed a shift in the intestinal microbial beta diversity (especially fungal beta diversity) of piglets with the oral administration of *L. frumenti* (**Figures 6A,B**), which is consistent with the results from weighted UniFrac cluster tree analysis (**Figures 6C,D**). To further dissect the alteration of intestinal microbial communities in piglets with oral administration of *L. frumenti*, we evaluated the alpha diversity in microbial communities using Chao index, Shannon index, and Simpson index, respectively. The results demonstrated that the intestinal bacterial alpha diversity was not significantly changed by *L. frumenti* administration, whereas the intestinal fungal alpha diversity was significantly changed by *L. frumenti* administration (**Figures 6E–J**).

**FIGURE 7:**
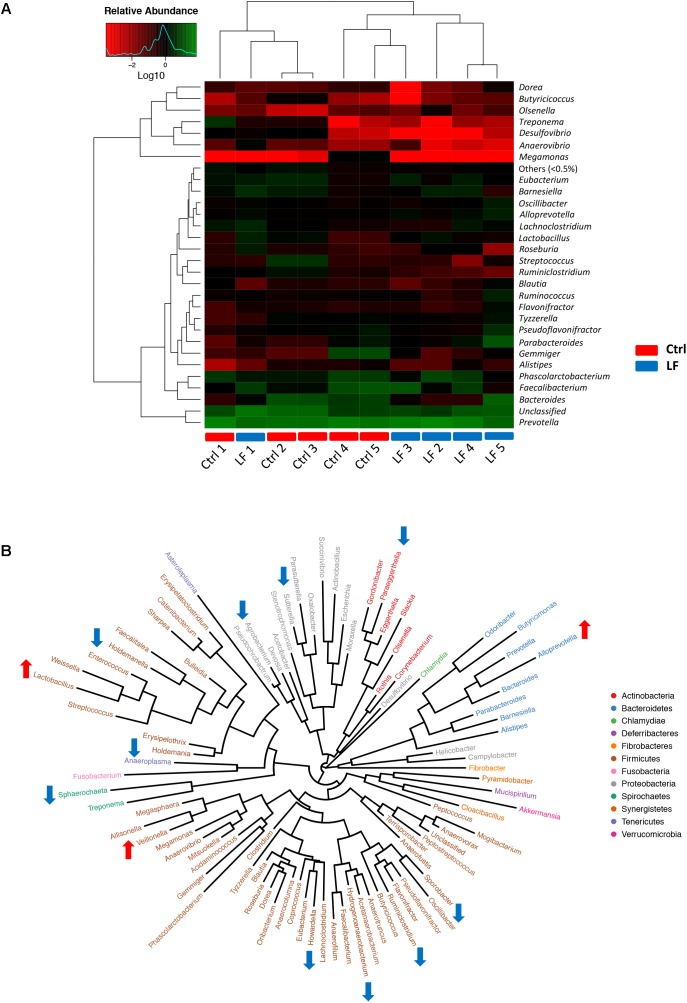
The alterations in intestinal bacterial taxonomic compositions at genus level in early-weaned piglets with oral administration of *L. frumenti*. **(A)** Heat map and hierarchical clustering of genera in the intestinal bacterial communities of early-weaned piglets. The values of color in the heat map represent the normalized relative abundances of genera (Log 10) (Ctrl, Control; LF, *L. frumenti*). **(B)** Phylogenetic tree was constructed from the genera identified in the intestinal bacterial communities of early-weaned piglets. Up arrow indicated that the relative abundance of the corresponding genus significantly increased with oral administration of *L. frumenti*, whereas down arrow indicated that the relative abundance of the corresponding genus significantly decreased with oral administration of *L. frumenti*. Metastats analysis was applied to identify the significantly differentially abundant bacterial genera among groups and detailed data were presented in the Supplementary Data S1.

**FIGURE 8:**
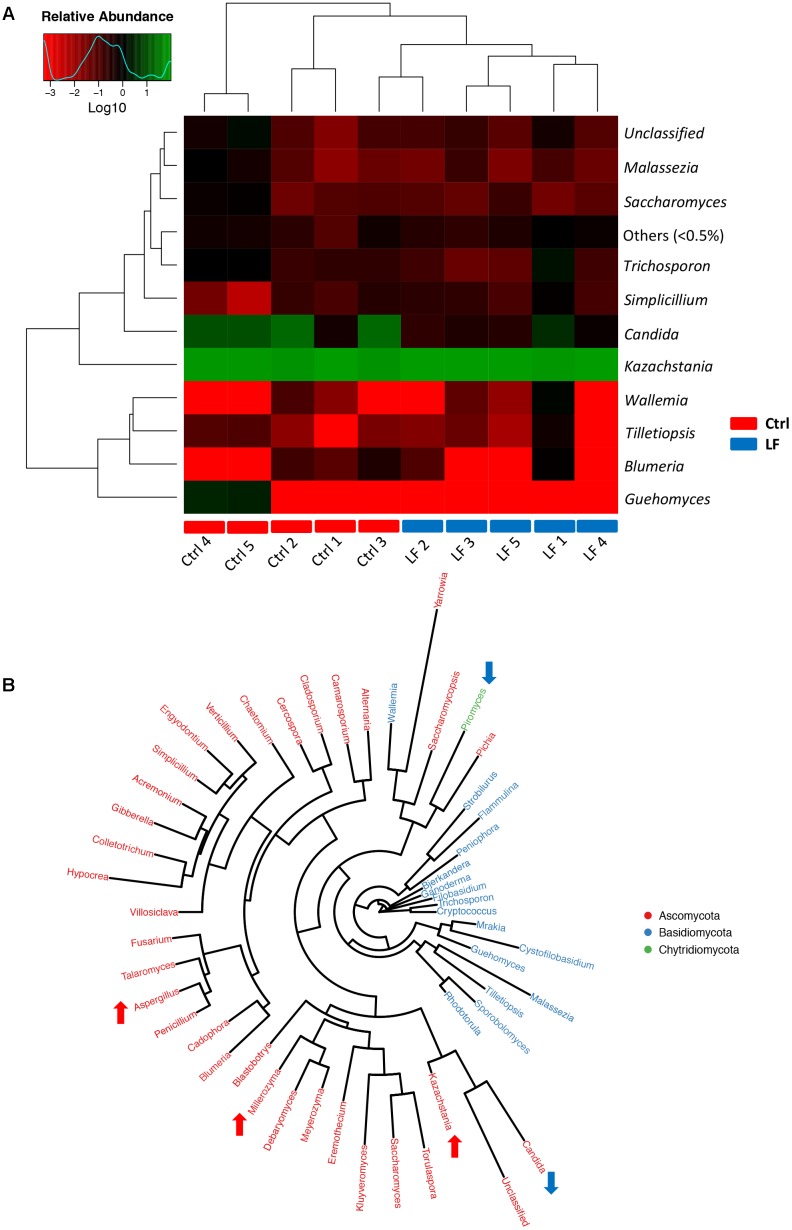
The alterations in intestinal fungal taxonomic compositions at genus level in early-weaned piglets with oral administration of *L. frumenti*. **(A)** Heat map and hierarchical clustering of genera in the intestinal fungal communities of early-weaned piglets. The values of color in the heat map represent the normalized relative abundances of genera (Log 10) (Ctrl, Control; LF, *L. frumenti*). **(B)** Phylogenetic tree was constructed from the genera identified in the intestinal fungal communities of weaned piglets. Up arrow indicated that the relative abundance of the corresponding genus significantly increased with oral administration of *L. frumenti*, whereas down arrow indicated that the relative abundance of the corresponding genus significantly decreased with oral administration of *L. frumenti*. Metastats analysis was applied to identify the significantly differentially abundant fungal genera among groups and detailed data were presented in the Supplementary Data S2.

To further investigate the shifts in taxonomic compositions of early-weaned piglets by *L. frumenti* administration, a total of 91 bacterial genera and 49 fungal genera were identified, respectively. These were 28 abundant bacterial genera (relative abundance > 0.5%) and 10 abundant fungal genera (relative abundance > 0.5%) presented in the intestinal microbiota of weaned piglets (**Figures 7A**, **8A**). Genus *Prevotella* and genus *Kazachstania* were the most abundant genera in the intestinal bacterial communities and fungal communities, respectively (**Figures 7A**, **8A**). The genus-level cluster analysis using heat map revealed a higher similarity of the samples within group than that among groups and a shift in intestinal microbial genus-level compositions (especially fungal genus-level compositions) by *L. frumenti* administration (**Figures 7A**, **8A**). We used Metastats analysis to dissect the alteration of intestinal microbial communities in piglets with oral administration of *L. frumenti*. The results demonstrated that the relative abundances of 3 bacterial genera (*Alloprevotella*, *Veillonella*, and *Lactobacillus*) significantly increased by oral administration of *L. frumenti*, while the relative abundances of 10 bacterial genera (including *Paraeggerthella*, *Sutterella*, *Howardella*, *Holdemania*, *Ruminiclostridium*, *Hydrogenoanaerobacterium*, *Holdemanella*, *Sporobacter*, *Sphaerochaeta*, and *Agrobacterium*) significantly decreased by *L. frumenti* administration (**Figure 7B**). Our data indicated that the relative abundances of three fungal genera (*Kazachstania*, *Millerozyma*, and *Aspergillus*) significantly increased and the relative abundances of two fungal genera (*Candida* and *Piromyces*) significantly decreased with *L. frumenti* administration (**Figure 8B**).

**FIGURE 9:**
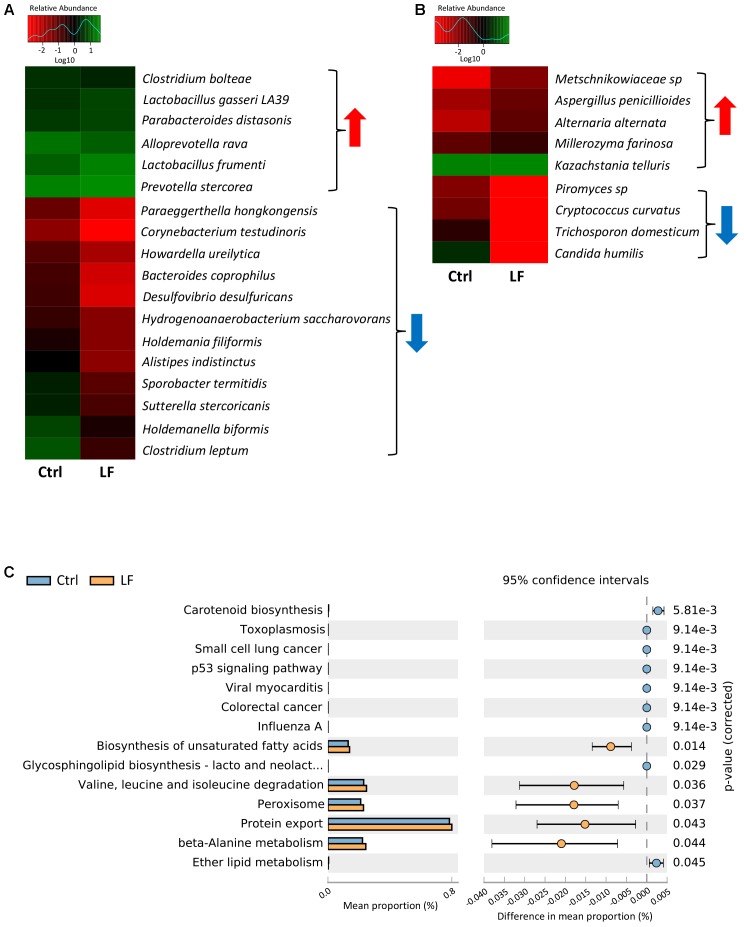
Significant shifts in intestinal microbial compositions at species levels and bacterial functional profiles in early-weaned piglets with oral administration of *L. frumenti*. **(A,B)** Heat map of differentially abundant intestinal bacterial species **(A)** and fungal species **(B)** in weaned piglets between the Ctrl group and LF group. The values of color in the heat map represent the normalized relative abundances of species (Log 10). Metastats analysis was applied to identify the significantly differentially abundant bacterial species and fungal species among groups and detailed data were presented in the Supplementary Datas S3, S4, respectively (Ctrl, Control; LF, *L. frumenti*). **(C)** The comparative analysis for the relative abundances of bacterial KEGG pathways between the Ctrl group and LF group. STAMP analysis was applied to identify the significantly differentially abundant KEGG pathways among groups and detailed data were presented in the Supplementary Data S5.

To further investigate the shifts of the taxonomic compositions of intestinal microbiota at species level with the oral administration of *L. frumenti*, a total of 147 bacterial species and 75 fungal species were identified in the microbial populations, respectively. Among them, the relative abundances of six bacterial species (*Clostridium bolteae*, *L. gasseri* LA39, *Parabacteroides distasonis*, *Alloprevotella rava*, *L. frumenti*, and *Prevotella stercorea*) significantly increased and the relative abundances of 12 bacterial species significantly decreased with oral administration of *L. frumenti* (**Figures 9A**). In fungal community, the relative abundances of five species (*Metschnikowiaceae* sp., *Aspergillus penicillioides*, *Alternaria alternata*, *Millerozyma farinosa*, and *Kazachstania telluris*) significantly increased and the relative abundances of four species (*Piromyces* sp., *Cryptococcus curvatus*, *Trichosporon domesticum*, and *Candida humilis*) significantly decreased with oral administration of *L. frumenti* (**Figure 9B**). Importantly, our data revealed that oral administration of *L. frumenti* significantly increased the relative abundances of health-promoting microbes (including *L. frumenti* (Ventura et al., 2009; van Baarlen et al., 2013), *L. gasseri* LA39 (Ventura et al., 2009; van Baarlen et al., 2013), *P. distasonis* (Kverka et al., 2011), and *K. telluris* (Ashraf and Shah, 2014; Sharma and Devi, 2014) and decreased the relative abundances of opportunistic pathogens [including *Desulfovibrio desulfuricans* (Hagiwara et al., 2014) and *C. humilis* (Netea et al., 2015; Rodrigues et al., 2016)].

To investigate the functional profiles of intestinal bacterial community, we used PICRUSt to predict the gene family abundances of bacterial communities based on the 16S rDNA gene data and a database of reference genomes as previously described (Langille et al., 2013). The results demonstrated that the relative abundances of the genes involved in fatty acids and protein metabolism (including the biosynthesis of unsaturated fatty acids, valine, leucine, and isoleucine degradation, protein export, and beta-Alanine metabolism) were significantly increased by *L. frumenti* administration, suggesting a significantly enhanced nutrients digestive system (**Figure 9C**). However, the proportions of the genes for some diseases-associated KEGG pathway (including toxoplasmosis, small cell lung cancer, viral myocarditis, colorectal cancer, influenza A) were significantly decreased by *L. frumenti* administration, suggesting a potential reduction in the risk of host diseases (**Figure 9C**). These findings above suggested that *L. frumenti* contributes to improve the intestinal microbial barrier function in early-weaned piglets.

## Discussion

In this study, we firstly report an important role of *L. frumenti* in the intestinal epithelial barrier functions improvement in early-weaned piglets, suggesting that *L. frumenti* may be a potential antibiotic alternative used in intestinal epithelial barrier functions damage prevention. Currently, the intestinal microbiota has been regarded as a “microbial organ” functioning in nutrient absorption and metabolism (Backhed et al., 2007), the host immune defense system development (Ivanov et al., 2009), the intestinal epithelium differentiation (Sommer and Backhed, 2013), and intestinal epithelial barrier function maintenance (Garrett et al., 2010). Interestingly, probiotics have shown efficacy in the maintenance of intestinal epithelial barrier function in mammals (Bron et al., 2011, 2017; Wan et al., 2016). *Lactobacillus* have been demonstrated be a class of probiotics which have health-promoting roles in mammals (Kleerebezem et al., 2010; van Baarlen et al., 2013). Our study demonstrated that *L. frumenti* have the role of intestinal epithelial barrier functions improvements in piglets for the first time. Thus, these findings further the understanding of health-promoting roles for *Lactobacillus.*

The improvement in intestinal epithelial barrier functions may be a result of improved intestinal epithelial physical barrier function, immunological barrier function, and intestinal microbial barrier function in early-weaned piglets. Intestinal epithelial tight junction proteins contribute to force necessary for maintenance of cell–cell interactions and primarily determine the paracellular permeability (Steed et al., 2010). Recent studies have showed that some probiotics (including *Streptococcus thermophilus*, *L. acidophilus*, *Bifidobacterium infantis*, and *Escherichia coli* Nissle 1917) contribute to the intestinal epithelial physical barrier function maintenance (Resta-Lenert and Barrett, 2003, 2006; Ukena et al., 2007; Zyrek et al., 2007; Ewaschuk et al., 2008) by increasing the intestinal epithelial tight junction proteins expression. Our data also demonstrated that oral administration of *L. frumenti* significantly improved the intestinal epithelial physical barrier function as evidenced by significantly increased intestinal epithelial tight junction proteins (including Occludin, Claudin-1, and ZO-1) expression. Mucins proteins secreted from the intestinal goblet cells form the mucous layer (McGuckin et al., 2011). The mucous layer can shield the epithelium from potentially harmful antigens and molecules, thereby acting as the important chemical barrier against the intestinal epithelial infection by pathogens (McGuckin et al., 2011; Johansson and Hansson, 2016). Previous studies showed that some *Lactobacillus* (Mattar et al., 2002; Mack et al., 2003; Kim et al., 2008) significantly increased the intestinal mucin 2 and mucin 3 expression. Probiotic mixture VSL#3 contributed to the intestinal mucin 1, mucin 2, and mucin 3 expression (Caballero-Franco et al., 2007). However, our data demonstrated that *L. frumenti* administration had no significant effect on the intestinal epithelial mucins expression which is consistent with the results of that a probiotic (*Escherichia coli* Nissle 1917) had no effect on the intestinal mucins expression in a previous study (Otte and Podolsky, 2004). These results suggested that potential probiotics may confer different effects on intestinal mucins proteins regulations and even the intestinal microbes belonged to same genus (such as *Lactobacillus*) may have distinct roles in intestinal mucins proteins regulations. Growing evidences have demonstrated that low pH in intestinal contents facilitate the intestinal homeostasis maintenance (van Baarlen et al., 2013). Our data demonstrated that pH values in intestinal contents in early-weaned piglets were not significantly changed by *L. frumenti* administration. These findings suggest that there is no significant alteration in intestinal epithelial chemical barrier function in early-weaned piglets with *L. frumenti* administration.

The intestinal epithelial immunological barrier functions have important role in the prevention for intestinal epithelium infection by pathogens (Rescigno, 2011). Our data demonstrated that oral administration of *L. frumenti* significantly improved the intestinal epithelial immunological barrier functions as evidenced by significantly increased serum IgG levels, intestinal sIgA levels, and intestinal IFN-γ levels. These data revealed that oral administration of *L. frumenti* contributes to the intestinal mucosal immunity performed by sIgA and anti-infection immunity performed by IgG and antiviral immunity performed by IFN-γ. Recent evidences have demonstrated that some probiotics [including *L. casei* (Ogawa et al., 2001; Galdeano and Perdigon, 2006), *Bifidobacterium animalis* (Martins et al., 2009), *Bifidobacterium bifidum*, *Bifidobacterium infantis* (Qiao et al., 2002), *Bifidobacterium lactis* (Shu and Gill, 2001), *L. helveticus* (Leblanc et al., 2004), *Saccharomyces boulardii* (Rodrigues et al., 2000; Qamar et al., 2001), *L. acidophilus*, and *Bifidobacterium bifidum* (Shandilya et al., 2016)] facilitate increasing the host sIgA levels and some probiotics [including *Bifidobacterium bifidum* (Tanaka et al., 2017) and *Bacillus subtilis* (Guo et al., 2017)] contribute to increase the host serum IgG levels. Thus, our findings facilitate our increasing understandings of the regulatory role of probiotics in host immune system.

Our results showed that oral administration of *L. frumenti* not only significantly increased the relative abundance of *L. frumenti*, but also altered the intestinal microbial community in the feces of early-weaned piglets as evidenced by altered microbial diversity, microbial taxonomic composition, and bacterial functional profiles. Our data revealed that oral administration of *L. frumenti* significantly increased the relative abundances of health-promoting microbes [including *L. frumenti* (Ventura et al., 2009; van Baarlen et al., 2013), *L. gasseri* LA39 (Ventura et al., 2009; van Baarlen et al., 2013), *P. distasonis* (Kverka et al., 2011), and *K. telluris* (Ashraf and Shah, 2014; Sharma and Devi, 2014)] and decreased the relative abundances of opportunistic pathogens [including *D. desulfuricans* (Hagiwara et al., 2014) and *C. humilis* (Netea et al., 2015; Rodrigues et al., 2016)]. These results suggested that *L. frumenti* may provide optimal growth conditions for those up-regulated intestinal microbes and inhibit the growth of those down-regulated intestinal microbes by some microbial products (such as bacteriocin, short-chain fatty acid, lactic acid, and other secondary metabolites). Our previous study revealed that functional maturation of the intestinal bacterial community was characterized by the significantly increased digestive system, glycan biosynthesis and metabolism, and vitamin B biosynthesis during the early period after weaning as the piglets aged, suggesting that enhanced intestinal microbes-mediated nutrients digestive system is vital for early-weaned piglets (Hu et al., 2016). Interestingly, our data suggest that functional alteration of the intestinal bacterial community by *L. frumenti* administration was characterized by the significantly increased fatty acids and protein metabolism (including the biosynthesis of unsaturated fatty acids, valine, leucine, and isoleucine degradation, protein export, and beta-Alanine metabolism), suggesting an enhanced intestinal microbes-mediated nutrients digestive function. *L. frumenti* administration also significantly decreased the diseases-associated metabolic pathways (including toxoplasmosis, small cell lung cancer, viral myocarditis, colorectal cancer, and influenza A) in intestinal bacterial community, suggesting that *L. frumenti* administration facilitates the intestinal bacterial community homeostasis and thus may decrease the risk of host diseases. Thus, oral administration of *L. frumenti* facilitate the functional maturation of the intestinal bacterial community in early-weaned piglets.

In sum, this study reveal that *L. frumenti* contributes to improve the intestinal epithelial barrier functions in early-weaned piglets. Thus, our findings provide a potential therapy for mammals at risk of intestinal epithelial barrier functions disorders.

## Ethics Statement

The animal experiments were carried out in strict accordance with the protocols (permit number: HZAUSW2013-0006) approved by the Animal Care and Use Committee of Huazhong Agricultural University. The animal care and maintenance were in compliance with the recommendations in the Regulations for the Administration of Affairs Concerning Experimental Animals of China.

## Author Contributions

JH and XY designed the research. JH, LC, WZ, MS, LL, CX, XW, YN, QH, XX, BX, YT, SZ, YY, TY, and LM conducted the research. JH and XY wrote the paper with the help of all authors. All authors read and approved the final version of the manuscript.

## Conflict of Interest Statement

The authors declare that the research was conducted in the absence of any commercial or financial relationships that could be construed as a potential conflict of interest.
