# Supplementary material for: Lactobacillus frumenti Facilitates Intestinal Epithelial Barrier Function Maintenance in Early-Weaned Piglets
Source: Front Microbiol. 2018 May 11;9:897. doi: 10.3389/fmicb.2018.00897 (PMC5958209; doi:10.3389/fmicb.2018.00897)
Supplement: Supplementary file 1 [file Table_1.PDF]

Supplementary Table 1 The main components in the diet for weaned piglets.

Components	Proportion
Crude protein	≥ 20%
Crude fibre	≤ 4%
Crude ash	≤ 8%
Total P	≥ 0.5%
Ca	0.5% ~ 1.2%
NaCl	0.4% ~ 1.4%
Lys	≥ 1.3%
