# Supplementary material for: Lactobacillus frumenti Facilitates Intestinal Epithelial Barrier Function Maintenance in Early-Weaned Piglets
Source: Front Microbiol. 2018 May 11;9:897. doi: 10.3389/fmicb.2018.00897 (PMC5958209; doi:10.3389/fmicb.2018.00897)
Supplement: Supplementary file 2 [file Presentation_1.PDF]

Supplementary Materials Legends

Supplementary Table 1 | The diet compositions for early-weaned piglets.

Supplementary Data 1 | Detailed data for Metastats analysis of intestinal bacterial genera.

Supplementary Data 2 | Detailed data for Metastats analysis of intestinal fungal genera.

Supplementary Data 3 | Detailed data for Metastats analysis of intestinal bacterial species.

Supplementary Data 4 | Detailed data for Metastats analysis of intestinal fungal species.

Supplementary Data 5 | Detailed data for STAMP analysis of KEGG pathways.
